# Biomimetic Scaffolds—A Novel Approach to Three Dimensional Cell Culture Techniques for Potential Implementation in Tissue Engineering

**DOI:** 10.3390/nano14060531

**Published:** 2024-03-16

**Authors:** Tomasz Górnicki, Jakub Lambrinow, Afsaneh Golkar-Narenji, Krzysztof Data, Dominika Domagała, Julia Niebora, Maryam Farzaneh, Paul Mozdziak, Maciej Zabel, Paweł Antosik, Dorota Bukowska, Kornel Ratajczak, Marzenna Podhorska-Okołów, Piotr Dzięgiel, Bartosz Kempisty

**Affiliations:** 1 Division of Histology and Embryology, Department of Human Morphology and Embryology, Wroclaw Medical University, 50-368 Wroclaw, Poland; jakub.lambrinow@student.umw.edu.pl (J.L.); maciej.zabel@umw.edu.pl (M.Z.); piotr.dziegiel@umw.edu.pl (P.D.); 2 Prestage Department of Poultry Science, North Carolina State University, Raleigh, NC 27607, USA; pemozdzi@ncsu.edu (P.M.); 3 Division of Anatomy, Department of Human Morphology and Embryology, Wroclaw Medical University, 50-368 Wroclaw, Poland; krzysztof.data@umw.edu.pl (K.D.); dominika.domagala@umw.edu.pl (D.D.); julia.niebora@umw.edu.pl (J.N.); 4 Fertility, Infertility and Perinatology Research Center, Ahvaz Jundishapur University of Medical Sciences, Ahvaz P.O. Box 6193673111, Iran; farzaneh-m@ajums.ac.ir; 5 Department of Veterinary Surgery, Institute of Veterinary Medicine, Nicolaus Copernicus University in Torun, 87-100 Torun, Poland; pantosik@umk.pl (P.A.); kornel@umk.pl (K.R.); 6 Department of Diagnostics and Clinical Sciences, Institute of Veterinary Medicine, Nicolaus Copernicus University in Torun, 87-100 Torun, Poland; dbukowska@umk.pl; 7 Division of Ultrastructure Research, Department of Human Morphology and Embryology, Wroclaw Medical University, 50-368 Wroclaw, Poland; marzenna.podhorska-okolow@umw.edu.pl; 8 Physiology Graduate Faculty, North Carolina State University, Raleigh, NC 27613, USA; 9 Department of Obstetrics and Gynecology, University Hospital and Masaryk University, 602 00 Brno, Czech Republic

**Keywords:** biomimetic scaffolds, 3D bioprinting, biologically derived materials, 4D materials

## Abstract

Biomimetic scaffolds imitate native tissue and can take a multidimensional form. They are biocompatible and can influence cellular metabolism, making them attractive bioengineering platforms. The use of biomimetic scaffolds adds complexity to traditional cell cultivation methods. The most commonly used technique involves cultivating cells on a flat surface in a two-dimensional format due to its simplicity. A three-dimensional (3D) format can provide a microenvironment for surrounding cells. There are two main techniques for obtaining 3D structures based on the presence of scaffolding. Scaffold-free techniques consist of spheroid technologies. Meanwhile, scaffold techniques contain organoids and all constructs that use various types of scaffolds, ranging from decellularized extracellular matrix (dECM) through hydrogels that are one of the most extensively studied forms of potential scaffolds for 3D culture up to 4D bioprinted biomaterials. 3D bioprinting is one of the most important techniques used to create biomimetic scaffolds. The versatility of this technique allows the use of many different types of inks, mainly hydrogels, as well as cells and inorganic substances. Increasing amounts of data provide evidence of vast potential of biomimetic scaffolds usage in tissue engineering and personalized medicine, with the main area of potential application being the regeneration of skin and musculoskeletal systems. Recent papers also indicate increasing amounts of in vivo tests of products based on biomimetic scaffolds, which further strengthen the importance of this branch of tissue engineering and emphasize the need for extensive research to provide safe for humansbiomimetic tissues and organs. In this review article, we provide a review of the recent advancements in the field of biomimetic scaffolds preceded by an overview of cell culture technologies that led to the development of biomimetic scaffold techniques as the most complex type of cell culture.

## 1. Introduction

Tissue damage resulting from cancer, congenital defects, and trauma requires new and effective treatments that facilitate tissue regeneration. Tissue engineering presents significant potential in this regard, as it enables the restoration of native tissue architecture and functions through the fusion of cells to specific scaffolds [1]. The goal of tissue engineering (TE) is to restore, preserve, or enhance the structure and function of damaged tissues or organs by integrating biological signals and biological scaffolding strategies [2].

Cell cultures have traditionally been studied on 2D platforms, consisting of cells interacting with a culture dish in a medium environment. However, this method has limitations as the growth of cells on a flat surface does not accurately reproduce their actual functioning in the 3D environment of other cells. The 3D model overcomes these limitations by recreating the in vivo environment. Scientists have provided evidence that scaffolding animal cells can induce the formation of 3D colonies that resemble the natural environment, both molecularly and phenotypically [3]. The extracellular matrix (ECM) plays a crucial role in the transport properties, cell communication, mechanotransduction, and growth factor signaling of the 3D hierarchical microstructure due to its electromechanical nature. This is achieved through the interaction of the ECM with receptors on the cell surface, as well as the binding of growth factors and other signaling molecules [4]. Zhang et al. suggest that the ECM scaffold is a highly promising candidate for tissue engineering applications. Among biomimetic ECM scaffold materials, decellularized ECM scaffolds (dECMs) derived from natural ECM are particularly noteworthy due to their natural components and microenvironment [5].

The goal of regenerative medicine is to repair and replace damaged tissue, and the use of three-dimensional scaffolds is one of the most promising techniques for tissue repair. Hydrogels are one of the most extensively researched types of scaffolds. They have demonstrated positive results in preclinical studies by mimicking the fundamental signals that promote local tissue regeneration [6]. Recent advancements in computer-aided design and 3D printing have helped the production of macroporous hydrogels, enabling the creation of more intricate structures. This approach may lead to the development of fully reconstructed organs. The field of hydrogels is a promising area of research in regenerative medicine, with applications in most tissues of the human body [7].

Biocompatible scaffolds promote cell adhesion, proliferation, and differentiation to facilitate tissue regeneration. These scaffolds provide strength, mechanical stability, flexibility, and an ideal environment for cell growth. Scaffolds can be divided into natural and synthetic categories [5]. Bioscaffolds provide a niche for cell growth, while synthetic scaffolds offer greater control over the size and morphology of regenerated tissues. In this review, we present a comprehensive overview of the current state of knowledge regarding biomimetic scaffolds. We begin by examining recent advancements in scaffold technology and their pivotal role in tissue engineering. Subsequently, we delve into the cell culture techniques that have contributed to the conceptualization and development of biomimetic scaffolds, as well as those evolving in tandem with this technology. In the final section, we highlight recent advancements in biomimetic scaffolds and explore their potential applications in biotechnology and tissue engineering.

## 2. Scaffolds in Tissue Engineering—Recent Findings and Current Research

In recent years, there has been an observable increase in the diversity of areas studied in the field of tissue engineering. Scientists are seeking more complex opportunities to apply recent technological advancements beyond the classic tissue engineering of the skeletal system [8]. An increasing number of studies provide data indicating usage of scaffolds in tissue engineering of different systems, including endocrine, muscle, genitourinary, digestive and pulmonary systems.

Recent advancements in regenerative endocrinology include the development of novel methods of protecting pancreatic β-cells from destruction by immune cells in hybrid devices consisting of scaffolds made out of polycaprolactone scaffolds and pancreatic β-cells encapsulated in alginate microcapsules. This method prolongs the functionality of the device and potentially eliminates the need for immunosuppressants [9]. Also, the development of prevascularized thyroid organoids opens new possibilities for potential new ways of hypothyroidism treatment [10]. Another advancement in tissue engineering of endocrine glands is development of a 3D-bioprinted functional model of the pancreas using pancreatic and endothelial cell lines with an ability to moderate insulin secretion answering to changes in glucose levels in medium [11]. Also, in the field of human reproductive hormones, there are some significant changes with the introduction of decellularized extracellular matrix providing optimal microenvironment for in vitro spermatogenesis [12].

In the treatment of volumetric muscle loss, scientists have developed a photoreactive hydrogel with the ability to change its stiffness. This allows the determination of the optimal level of hydrogel stiffness, which significantly increases the regeneration of muscle tissue [13].

In the field of muscle tissue regeneration, a recent study has also provided a description of complex artificial muscle tissue consisting of a layer of myofibroblasts connected with motor neurons derived from induced pluripotent stem cells. The motor neurons contracted the layer of myofibroblasts by creating neuromuscular junctions [14].

A study conducted on rabbits demonstrated another potential application of scaffolds in tissue engineering of different systems. It presents the potential usage of adipose tissue engineering as a tool in the treatment of postoperative complications. In order to reduce epidural fibrosis after the procedure of laminectomy, the researchers reconstructed local adipose tissue using a scaffold made from ECM imbued with mesenchymal stem cells [15].

Another area of tissue engineering development is tissue engineering of bile ducts using hydrogels as the scaffold [16].

Scientists have also recently used tissue engineering technologies in the regeneration of the genitourinary system. They created a device consisting of a scaffold and mesenchymal stem cells overexpressing basic fibroblast growth factor. They have provided evidence of the device’s potential usage in regenerating full-thickness injuries of the uterus [17].

Tissue engineering is a developing field that has generated interest in creating scaffold-based artificial organs with higher functionality. For instance, a recent study focused on contractile vascular grafts that retain their contractility. The graft is composed of decellularized pulmonary artery and progenitor cardiovascular cells obtained in a bioreactor under physiological flow conditions [18].

## 3. 2D Cell Cultures—Limitation of Most Common Type of Cell Cultures

**Figure 1:**
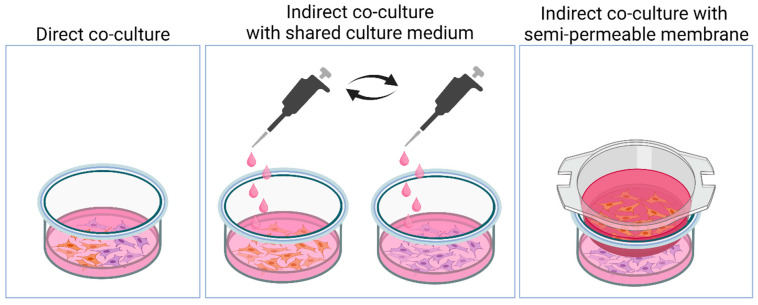
Types of direct and indirect co-culture systems. Created with BioRender.com.

Culture vessels with a single surface are used for 2D cell culture. The proliferating cells cover the surface of the culture environment, forming a monolayer cell culture. The crucial factor affecting cell proliferation is confluence, which refers to the degree of surface coverage by cells. As confluence approaches 100%, the metabolism of cultured cells changes and the expression of proliferation markers decreases [19]. The most commonly used technique for culturing cells is on a flat surface due to its simple processing and cost-effectiveness. A monolayer of cells also enables straightforward observation and measurement. There are two types of 2D cultivations: simple cultures and co-cultures. Simple culture involves the interaction of cells with a dish in a culture medium environment. In co-cultures, cells from different tissues are cultured together in a single medium, allowing for direct or indirect interactions, as illustrated in Figure 1. Direct co-culture involves growing different types of cells on the surface of a shared dish, facilitating plenty of interactions. Indirect co-culture, on the other hand, is based on separate cultivation surfaces but with interaction occurring through a common culture medium. One disadvantage of this 2D cultivation model is the lack of representation of real cell surroundings. Although growing cells on a flat, plastic surface covered with dry plasma can enhance cell adhesion, it is not an appropriate method for studying cell metabolism in a natural environment where cells are surrounded by other cells in three dimensions. However, this model is useful for analyzing simple interactions under controlled conditions.

## 4. 3D Cell Cultures

The next stage in comprehending physiological and pathological processes involving cell cultures is through the use of three-dimensional cell cultures. The interest of researchers in 3D human cell culture has grown rapidly since Hamburger and Salmon published one of the first papers on the subject in 1977 [20,21]. Although 2D cell cultures are the most commonly used method of studying living cells in vitro, there are still some significant drawbacks to this approach. According to research, cells cultured in two-dimensional conditions exhibit less similarity to those found in natural tissues [22]. This is due to changes in cell morphology, reduced cell–cell and cell–ECM interactions, and altered gene and protein expression resulting from the lack of a complex microenvironment [23]. Researchers were compelled to explore 3D culture techniques due to impaired cell polarity, ample access to oxygen and nutrition, and the absence of an external matrix, among other characteristics [24]. In the early 2000s, it became evident that 3D cultures were an emerging technology [25]. While the prediction that 3D culture would replace 2D culture was somewhat misguided, there is a growing trend towards more studies utilizing 3D culture. Three-dimensional cell cultures offer several advantages, the most significant of which is a closer representation of natural tissues [26]. However, 3D cultures also have some disadvantages, including higher costs, lower reproducibility, and greater difficulty in interpretation [23].

In fact, three-dimensional cell culture is a more complex method than two-dimensional culture. 3D cultures often involve co-culturing, where more than one type of cell is cultured simultaneously. In addition, there are various methods for replicating living 3D structures. 3D culture can be categorized as either scaffold-free or scaffold-based [26,27,28,29]. In scaffold-free 3D cultures, cells interact with each other and form structures such as spheroids [30]. Scaffold-based 3D cultures are capable of creating more intricate structures by utilizing cell–cell and cell–EMC interactions, which can closely resemble natural tissue [31]. There are two main types of 3D culture methods: those that use scaffolding in the cell culture (scaffold techniques) and those that do not (scaffold-free techniques).

### 4.1. Scaffold Free Techniques

#### Spheroids

**Figure 2:**
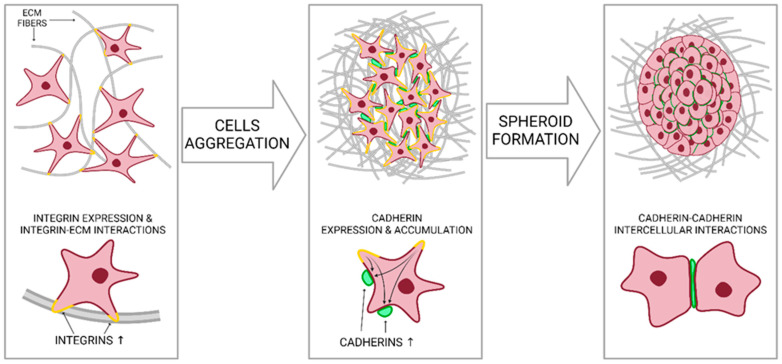
Steps of cell aggregation and spheroid formation (ECM, extracellular matrix). Created with BioRender.com.

The term ‘spheroid’ describes the spherical shape of cell colonies, whether they are single-cell or multicellular. In traditional monolayer cultures, cell–flask interactions are the primary type of interaction that contributes to the formation of the culture. In traditional monolayer cultures, cell–flask interactions are the primary type of interaction that contributes to the formation of the culture. However, spheroids emerge due to cell–cell and cell–ECM interactions. The process of forming a spheroid involves three main steps presented on Figure 2. Firstly, the extracellular matrix (ECM) fibers, which are rich in RGD motifs, allow for the binding of integrins expressed on the cell membrane surface, resulting in upregulated cadherin expression. Secondly, cadherins localize on the cell surface. Finally, cadherin-cadherin homophilic bindings between nearby cells cause stronger cell-to-cell adhesion and spheroid formation [24,32,33].

There are various methods for manufacturing spheroids. This goal can be achieved using various technologies, such as hanging drop, low-adherence substrate, bioreactor, microwells, magnetic manipulation, and microfluidics [33,34,35,36]. The properties of spheroids are more important than the technologies used to manufacture them. Due to their three-dimensional nature, spheroids closely resemble in vivo conditions, including oxygen gradients, nutrition, and metabolic products [35,37].

However, spheroids have some limitations despite their similarities to in vivo processes. This method of cell culture is less well-known than 2D monolayer cultures, and there are also major problems with standardizing spheroid size [33]. Nonetheless, spheroids are an important tool for understanding physiological and pathological processes. For instance, the proliferation rate of spheroid surface cells is significantly higher than that of the inner zone. In fact, due to the oxygen gradient, the inner zone may even consist of a necrotic core instead of living cells [35]. Therefore, spheroids are a superior model to 2D cultures for anti-cancer drug testing [38].

Currently, researchers are exploring the use of spheroids in various fields of bioengineering and medicine. This type of 3D culture is widely studied as an example of cells that mimic cancer tumors. In the case of co-cultures, which are spheroids made up of cancer cells and other cells such as cancer-associated fibroblasts, the similarities to in vivo cancer tumors are even greater [37]. The number of recently published papers on the use of spheroids in cancer research is still increasing. One of the main advantages of using spheroids in cancer research is the ability to measure drug response. Multiple studies have been conducted involving spheroids derived from various types of cancer, including colorectal cancer [39,40,41,42], breast cancer [41,43,44], lung cancer [45,46], ovarian cancer [1,47,48] and even Ewing sarcoma cells [49]. It is important to note that 3D cultures, particularly spheroids, are a valuable and effective tool for studying cancer biology. They can be used to assess potential drug resistance, identify new therapeutic targets, and investigate molecular mechanisms [50].

### 4.2. Scaffold Techniques

#### 4.2.1. Organoids

Organoids are more complex structures than spheroids and are named as such because they represent the complexity of organs, unlike spheroids, which generally represent only one type of tissue [30]. Organoids can be derived from various types of stem cells, including embryonic, induced pluripotent, or tissue-specific cells [24]. Due to their complexity, organoids are valuable models for studying and simulating physiological processes that occur in living organisms’ organs. Organoids are self-organising and self-renewing structures, meaning that they have some level of freedom in their organisation. While 2D cultures are easier to control, 3D cultures, especially more complex ones, are less predictable in terms of deterministic processes. Therefore, organoids can closely mimic living organs. Similarly, 3D cultures can be scaffold-based or scaffold-free [51]. However, scaffolds are more commonly used in organoid manufacturing to recreate a natural environment [52].

Organoids are often referred to as mini-organs, and current research is focused on creating fully functional organs on a mini-scale. Examples include ‘mini-guts’ [53], ‘mini-hearts’ [54], and ‘mini-livers’ [55]. The name ‘organoid’ accurately describes a miniaturised model of an entire organ. Organoids are commonly used in cancer research, with patient-derived organoids (PDOs) proving to be a valuable tool in drug testing and the study of cancer biology [56,57,58]. Additionally, organoids are utilised as models for a range of other diseases, including lung diseases [59], liver cirrhosis [60], inflammatory bowel disease [61], and brain disorders [62].

#### 4.2.2. Hydrogels

Modern 3D cell culture scaffold techniques often rely on the use of various biomaterials, including hydrogels. Hydrogels are hydrophilic, cross-linked polymer chains [63,64] that can be pre-prepared to facilitate 3D cell culture due to their similarity to the extracellular matrix [65]. Although hydrogels can vary greatly in water content, chain composition, and ionic charge [66], their use in 3D cell culture is well-established. The potential applications of hydrogels are vast, as demonstrated by the numerous classifications available. Hydrogels can be classified based on their source (natural or synthetic), size (nano-, macro-, or bulk hydrogels), chain composition, ionic charge, method of crosslinking, response to various stimuli, or biodegradability [67]. Matrigel^®^ is one of the most well-known hydrogels in scientific research [68]. The substance is a natural biomaterial derived from secretions of Engelbreth–Holm–Swarm (EHS) mouse sarcoma cells. Hydrogels can also be obtained naturally by crosslinking collagen [69], gelatin [70], hyaluronic acid [71], or alginate [72]. Synthetic hydrogels, in contrast to natural ones, can be manufactured with greater precision, allowing for better control of desired properties such as water absorption and ionic charge.

#### 4.2.3. Advanced 3D Bioprinting

3D bioprinting is a promising and increasingly popular tool for scaffold production, presenting many advantages. These include high levels of structure customization, which can imitate the structure of desired tissue, as well as the freedom to mix components and impregnate them with additional substances [73]. It allows for the creation of complex tissue structures that scientists around the world are looking to apply in medicine [74]. Current research primarily focuses on using 3D bioprinting to produce advanced scaffolds for skin bioengineering [75], as well as for musculoskeletal, cardiovascular, and neural systems [73].

The recent development of 3D bioprinting technologies has led to the creation of 4D bioprinting, which represents the next generation of biomaterials. These materials are capable of changing their shape, properties, and functions in response to external stimuli such as heat, changes in pH, light, and humidity [76]. A wide range of intelligent polymers and materials are used in this process. For example, iron-sensitive hydrogels, polyethylene glycol that responds to temperature fluctuations, or special ink containing poly(lactic acid) polymer that assembles into tubular shapes under the influence of a magnetic field [77]. This new generation of biomaterials is proposed to have applications in various areas of medicine, such as drug delivery systems (encapsulation devices) and biosensors [78,79,80]. However, the most significant potential of 4D-printed biomaterials is in tissue engineering [76]. Multi-material 4D-printed implants with heterogeneous morphology can provide an environment that changes over time that promotes cell activity and differentiation, and increases the regenerative capabilities of nearby tissues [81]. Currently, scientists are conducting research on 4D-printed biomaterials in a few applications. This includes the production of functional meniscal implants [82], regeneration of cartilage with chitosan derivatives [83], bone replacement implants for treating losses caused by trauma or genetic diseases [84,85,86,87], and tooth implants [88]. Additionally, there is evidence of the potential usage of 4D-printed biomaterials in the engineering of the cardiovascular system. Scientists are currently developing 4D-printed patches to aid heart function after myocardial infarction [89], as well as a model of an artery to study the pathomechanism of cardiovascular diseases [90]. Additionally, there are projects focused on creating 4D-printed heart valve implants [91]. 4D-printed biomaterials may also have applications in otolaryngology, such as craniofacial, tracheal, nasal, and aural implants [77]. Studies have shown that 4D-printed biomaterials can successfully guide stem cell differentiation and fate [92]. The potential applications of 4D-printed biomaterials include regeneration in craniofacial skeletal muscle [93], wound healing [94], implants for bladder disorders [95], and in vitro models of fibroblast remodeling [96].

Bioprinting techniques can be divided into two categories based on the place of synthesis. The most common technique is ex vivo, where the scaffold is combined with cells and other biomolecules outside of the body [76]. The newer approach, in situ, aims to print tissue or organs directly in the human body at the required site of trauma [97]. This technology has a wide variety of potential applications. There are various materials that are considered for use in in situ bioprinting, such as hydrogels based on Gallol-Functionalized Hyaluronic Acid [98], methacrylate-based gelatin [99], and bioinks based on alginate–chitosan and kaolin [100]. Current research focuses on in situ bioprinting applications for direct cartilage repair [101,102] or bioprinting human mesenchymal cells that will transdifferentiate into chondrogenic tissue [103]. In situ bioprinting may also be used for skin wound regeneration, providing accurate coverage of the affected area [104]. Scientists have developed a 3D bioprinter called the ‘SkinPen’ for skin regeneration, which uses a complex hydrogel controlled by ultrasound and ultraviolet light to enhance adhesive and morphological properties [105]. Currently, studies are being conducted to combine these two goals and create a robot-assisted in situ bioprinter for skin and hair follicle regeneration [106]. The study investigates the use of gelatin methacryloyl (GelMA) with zinc and silicon ions for hair follicle regeneration in a mouse model [107]. Additionally, researchers are exploring the use of robotic technologies for cranial bone regeneration through in situ bioprinting [108]. Robots are also being utilized to develop minimally invasive bioprinting systems for liver tissue bioprinting and regeneration [109]. In endoscopic surgery, in situ bioprinting is seen as a potential new technology for intestine regeneration [110]. Scientists have presented evidence of the potential use of in situ bioprinting for printing neurons, vascular and muscle tissues [111,112,113]. Intraoperative in situ bioprinting can also be used in orthopaedics for bone tissue regeneration [114]. Additionally, researchers are attempting to bioprint bone tissue with complete vascularisation, adding an additional level of complexity to the constructs created in situ [115]. Scientists see another potential application of in situ bioprinting in functional segmental tracheal reconstruction, where this technique can be used to regenerate destroyed parts of the trachea [116]. In addition, in situ bioprinting may have possible applications in dentistry as a means of regenerating dental pulp [117].

#### 4.2.4. Alternative Materials Employed in the Context of 3D Bioprinting

**Figure 3:**
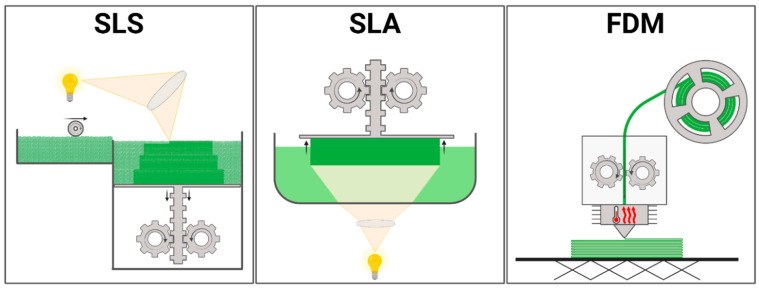
Main 3D printing technologies (abb. SLS, stereolitography; SLA, selective laser sintering; FDM, fused deposition modeling). Created with BioRender.com.

The three most commonly used techniques in 3D printing are Fused Deposition Modeling (FDM), Stereolithography (SLA), and Selective Laser Sintering (SLS). The crucial difference between these methods is the form of the delivered material and the way the printing material hardens, as shown in Figure 3. FDM is the most commonly used technique, where a solid filament is heated, melted, and extruded through a nozzle. The base of the SLA technique is a container with liquid resin that is hardened with a laser beam. Printing with SLS technique also involves a laser beam, but the printing material used is polymeric powder. The materials can be modified and supplemented in various ways due to the printing technique.

**Fused Deposition Modeling (FDM): FDM** is a method commonly used for 3D printing with several thermoplastic polymers, including poly (L-lactic acid) (PLA), poly-l-lactide-co-ε-caprolactone (PLCL), and gelatin methacrylate (GelMA), along with various supplements. PLA, which has stiff and cytocompatible mechanical properties, is most often used for bone tissue scaffolds [118]. PLA is obtained through the condensation of lactic acid or the polymerization of lactide. Commercially available polylactic acid (PLA) is typically derived from fermented plant starch, such as corn, cassava, sugarcane, or sugar beet pulp. However, PLA has limitations, including poor mechanical properties, insufficient surface wettability, and a low degradation rate, which restricts its use in biomedical applications [119]. Therefore, there are multiple proposals to enhance the PLA composition or indirectly use PLA for forming bioscaffolds. The use of a PLA printed shape as a frame for low-viscosity bioink can limit the ink from spreading beyond the established shape [120]. This strategy involves using PLA material as a scaffold to form the proper bioscaffold. To mimic tissues, particularly bone tissue, PLA is supplemented to improve biomineralization and physical properties. The addition of hydroxyapatite (HAp) significantly increases the strength and stiffness of PLA. Simultaneously, hydroxyapatite (HAp) contains sites for the deposition of salts and apatite species in the scaffold, which increases the rate of material mineralization in an in vitro study [121]. Bioscaffolds formed from HAp-polylactic acid (PLA) composites can improve bone regeneration in in vivo grafting. As demonstrated in the rabbit model, HAp-PLA scaffolds are biocompatible, degrade over time, and form bone trabeculae and marrow cavities on the surface of the scaffolds. Moreover, the safety and efficacy of HPa-PLA in repairing cranial defects in rabbits are comparable to that of autologous bone transplantation [122]. Bioglass (BG) can also be used to enrich the scaffold and mimic the bone tissue environment. The PLA-BG composite exhibits greater durability than PLA alone [123]. Furthermore, the addition of BG enhances cell viability and the expression of endothelial marker genes in vitro, as demonstrated on human umbilical vein endothelial cells (HUVECs), indicating a positive effect on angiogenesis [124]. Pearl powder [125], graphene oxide [126], and cold argon plasma treatment [119] are all valuable supplements that can influence bone cell differentiation, proliferation, and scaffold strength.

PLCL is a copolymer composed of L-lactic and ε-caprolactone. It is widely used in the production of scaffolds for soft tissue engineering due to its flexibility and rubber-like elasticity. With the adjustment of 3D-printing parameters, it is possible to obtain scaffolds with extensibility comparable to native human tissues, such as vessels, cartilage, and ligaments, while maintaining full cytocompatibility and cell adhesion [127]. A combination of PLA and PLCL has been found to be an effective mimic for cartilage tissue. The inclusion of PLA in the material enhances its mechanical resistance and stiffness, as well as the processability of PLCL for 3D printing. In line with this, the PLCL-PLA scaffold has been shown to improve the proliferation and chondrogenesis of in vitro-seeded chondrocytes more effectively than the PLCL scaffold alone [128]. The hybrid scaffold containing PLCL and decellularized extracellular matrix (dECM), specifically adipose tissue dECM, is a promising technique with potential clinical applications. These adipose-mimicking scaffolds possess mechanical properties comparable to native tissue and have the potential to enhance tissue regeneration. The in vitro test demonstrated that the dECM-PLCL scaffold promotes adipogenesis and angiogenesis, as well as adipose tissue formation, while suppressing apoptosis of human adipose-derived stem cells (hADSC) in vivo [129,130].

Gelatin methacryloyl (GelMA) is a hydrogel made from proteins. It has a porosity of almost 90% and a disordered pore arrangement, which imitates the structure of the extracellular matrix (ECM) due to its high collagen content. This porosity promotes cell viability and is crucial for proper development and metabolism [131]. To cross-link GelMA, it must be exposed to UV light in the presence of photo-initiators. The advantage of this process is its simplicity in modulating mechanical properties by adjusting the time and intensity of UV light and the concentration of photo-initiators. Commonly used photo-initiators include Irgacure 2959 (I2959), lithium phenyl-2,4,6-trimethylbenzoylphosphinate (LAP), and Eosin-Y [132]. However, a disadvantage of photo-crosslinking is the accelerated enzymatic degradation of the scaffold with collagenase, which makes the material less suitable for the regeneration process. The combination of GelMA and tyramine-conjugated 8-arm poly(ethylene glycol) (8PEGTA) reduces degradation while maintaining ECM-mimicking conditions during early tissue regeneration [133]. GelMA can also act as a carrier and support nervous tissue development. A 3D-printed canal filled with 7,8-dihydroxyflavone (7,8-DHF) successfully reconstructed and reconnected a 12-mm nerve defect [134]. The properties of elastic GelMA include the potential to mimic vessel structures. When supplemented with chitin nanocrystals, the material exhibits improved mechanical resistance, cell adhesion, proliferation, and vascularization [135]. GelMA can be used to mimic bone tissue. Supplementing with BG improves mechanical properties, cell adhesion, proliferation, and, most importantly, osteoblast differentiation. This also enhances the release of osteocalcin (OCN), a factor in bone mineralization that orchestrates the osteogenesis process [136].

**Stereolitography:** Compared to FDM printing, SLA printing is more efficient in printing smaller and more precise models with higher resolution. Stereolithography is a 3D printing process that uses a light source to harden the material. Even when using the same materials, such as GelMA, which is also used in SLA printing, the liquid state of the materials allows for the use of resins such as poly(D,L-lactide) (PDLLA) or poly(propylene fumarate) (PPF). Even when using the same materials, such as GelMA, which is also used in SLA printing, the liquid state of the materials allows for the use of resins such as poly(D,L-lactide) (PDLLA) or poly(propylene fumarate) (PPF).

Factory tests have demonstrated the possibility of utilising GelMA and a low-cost, commercially available printer to conduct three-dimensional in vitro culture. The scaffold produced in this manner is characterised by high printing accuracy and good biocompatible properties [137]. Similar to the FDM method, the enrichment of GelMA with other materials significantly expands and diversifies its potential uses, allowing it to be adapted to mimic various tissues. The combination of PLCL and GelMA has been shown to replicate the properties of small intestine tissue when cultured in vitro [138]. It is important to maintain a clear and logical structure, use precise language, and avoid bias and grammatical errors. Additionally, the supplementation of HAp has successfully aided in the regeneration of bone tissue in vivo [139]. Modifications using the FDM technique may also allow for the replication of other tissue types and material properties.

PDLLA is a flexible polymer with numerous medical applications, including controlled drug delivery [140]. It has a high capacity to mimic bone tissue and has been successfully used for bone regeneration in vivo. The best results in the treatment of bone defects were obtained using a hybrid of PDLLA and PPF. This hybrid also supported the expression of key markers for osteogenesis, such as osteocalcin, collagen, and runt-related transcription factor 2 (RUNX2) [141].

Photo-crosslinkable PPF is a popular choice for bone-mimicking scaffolds due to its superior mechanical resistance, stiffness, and biodegradability. To apply PPF, SLA is necessary using a solvent. Diethyl fumarate (DEF) is a commonly used solvent for PPF, but studies have shown that this combination can have a cytotoxic effect on seeded cells. Ethyl acetate (EA) is a potentially safer alternative. It has been found not to reduce scaffold biocompatibility on preosteoblasts cell-line in vitro [142]. By properly optimizing the PPF material composition, it is possible to create a drug-releasing bioscaffold with the strength and stiffness of native bone [143].

**Selective Laser Sintering:** Selective laser sintering is advantageous due to its ability to quickly and precisely print complex structures without the need for additional support. The printed elements are supported by powder, which also serves as a printing material. While there is limited research on the use of SLS as a source of scaffolds in medical applications, some materials have been described in this field. Borate-based bioactive glass was used to create bone-mimicking scaffolds that efficiently regenerate bone defects. A powder polymeric binder was used to merge the components, and after the fabrication process, the parts were heated to remove the binder [144]. One type of polymeric binder that can be used is thermoplastic polyurethane (TPU), which is also a suitable scaffold material. TPU is an elastic material with cytocompatible properties that could be used to mimic soft tissues. This enhances biological properties, maintains cell viability, promotes cell proliferation, and differentiates neural stem cells [145].

## 5. Biomimetic Scaffolds—From Advanced Engineering to Biological Application

**Figure 4:**
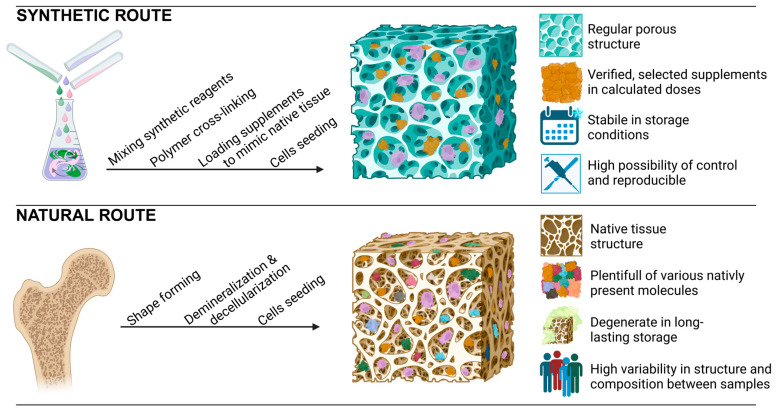
Synthetic and natural route of proceeding biomimetic scaffolds. Created with BioRender.com.

Biocomposite structures consist of macromolecules, such as proteins, lipids, polysaccharides, minerals, and polynucleotides, that are naturally present in tissues. The extracellular matrix (ECM) contains various types of proteins, including collagen, elastin, gelatin, and other glycoproteins. Collagen, which is abundant in connective tissues, forms a fibrillary structure that provides strength and structural support. The collagen fibers in the bone tissue are saturated with minerals, which increases their stiffness and mechanical strength. Bioactive domains in collagen are involved in interacting with cell membrane receptors, such as integrins (i.e., α1β1, α2β1, α10β1 and α11β1). Collagen-binding integrins influence fibroblast activity, regulating differentiation and synthesis of ECM components, which is crucial in tissue wound healing [146]. The presence of elastin in tissues provides elasticity to various organs, such as bladder and artery tissues. The mechanical properties of blood vessels, fibrous connective tissue, and skin are mainly determined by the cooperation of collagen and elastin percentage composition, which provides strength and structural support while also providing elasticity and resilience. Another crucial protein is fibronectin, which has domains that bind to other ECM proteins, such as collagen, heparin, and integrins. Interactions between cells and the extracellular matrix (ECM) play a crucial role in ECM development, homeostasis, and wound healing [147]. As extracting ECM proteins can be expensive, gelatin has been proposed as a cost-effective alternative scaffold base [148]. Polysaccharides, such as chitosan, alginate, dextran, and hyaluronic acid, have advanced scaffold development due to their low cost, ease of commercialization, biocompatibility, and biodegradability. They are similar to the extracellular matrix (ECM), which is rich in glycosaminoglycans, glycoproteins, and glycolipids [149,150,151,152]. Tissue engineering scaffolds are generally produced as pre-fabricated or in situ cross-linked hydrogels, with many using 3D printing technology. Pre-fabricated scaffolds primarily focus on the presence of pores and interconnected channels to enhance the viability of seeded cells. Figure 4 present 2 possible concepts of biomimetic scaffolds fabrication that employ various techniques, including traditional methods like decellularization but also electrospinning, rapid prototyping-based microfabrication, and modular hierarchical assembling. Each method provides different characteristics to porous scaffolds. These include degradability to nontoxic materials, production of ultrafine fibers with varying diameters, production of final 2D structures, and production of large and complex 3D structures based on 3D programmed images. In situ gelled hydrogel involves creating biomimetic hydrogel scaffolds using peptide-based biomaterials to repair tissue, as well as controlled drug delivery. Proteins possess molecular properties that enable them to interact with other macromolecules and regulate the hard and soft tissue of an organism.

The scaffold is a tool that creates space for tissue formation de novo. It mimics the natural environment to provide optimal conditions for cell growth. The properties of the culturing environments vary depending on the cultured tissue, not only with the material and composition of supplements but also with the mechanical aspects. Tissue engineering employs engineering and life science to generate biological structures to restore, repair, and maintain organ tissue and improve its function. Biological scaffolds can be made from native tissues or synthetically synthesized and can be either degradable or nondegradable [153]. The optimization of scaffold production relies on adjusting the material’s biological and chemical properties, such as biocompatibility, immunogenicity, and impact on cell metabolism [154]. The mechanical properties of the bioscaffold are equally important. The adjustment of material cross-linking density enables the regulation of porosity and resistance. A material with a denser cross-linking exhibits greater strength, tear resistance, and stiffness, which may be crucial for supporting connective tissue but can reduce cell viability [155]. Biomechanical resistance testing involves determining ultimate strain and stress and the energy required for failure, particularly under tensile forces [156]. Additionally, scaffolds have been utilized as controlled release tools to maintain therapeutic concentrations of diffusible tissue inductive factors. Loading a bioscaffold with the long-term released proangiogenic factor, leonurine hydrochloride (LH), can increase the osteogenic differentiation of bone marrow stem cells (BMSC) and promote vessel formation in vivo [157]. The long-term release of the anti-bacterial polyhexamethylenebiguanide (PHMB) efficiently reduces bacterial content in regenerating skin wounds [158]. Cell culture is a primary in vitro biological tool and a crucial component of transgenesis, bioengineering, and regenerative medicine. The extracellular matrix (ECM) plays a critical role in tissue structure and function. For example, tendons contain thick bundles of collagen type 1, which are responsible for their high tensile strength. Collagen and elastin fibrils are responsible for the elasticity of the skin. In terms of tissue function and activity, there are examples such as the Arg-Gly-Asp (RGD) sequence on fibronectin that triggers a binding event or heparin sulfate proteoglycans that facilitate basic fibroblast growth factor (bFGF) activities. ECM provides a degradable environment that is crucial for angiogenic sprouting and remodeling during tissue dynamics, including morphogenesis, homeostasis, and wound healing [159,160]. Embryonic ECM is replaced during morphogenesis to accommodate tissue growth, with an ECM half-life of 7 to 10 h [161]. After tissue development is complete, the physiological degradation of the ECM shifts to maintaining ECM homeostasis. Both morphogenesis and homeostasis strictly involve the p53/laminin pathway [162]. The p53 protein responds specifically to laminin, a key component of the ECM, and regulates the expression of molecules required to establish homeostatic form and dynamics [163]. The production of nitric oxide (NO), an important intercellular signaling molecule [164], also supports another link in the pathway.

Biomimetic scaffolds of the extracellular matrix (ECM) are designed to provide a framework for cell culture to develop tissue with appropriate signal cross-talk. Intercellular communication in 3D co-cultures is more efficient with the involvement of paracrine signaling [165]. An ideal biomimetic scaffold should imitate natural ECM properties and create conditions for positive interaction with cells to increase cell adhesion, growth, migration, and differentiation [166]. Decellularized extracellular matrix (dCEM) scaffolds contain extracellular macromolecules, including collagen, elastin, fibronectin, laminin, and matricellular proteins [158]. These scaffolds are prepared through a decellularization process, which preserves signals and biological performance, providing a 3D biological support structure for subsequent cell seeding on damaged organs. In the model of mice myocardial infarction, a bioengineered cardiac patch was created using decellularized tissue with seeded cells. This approach demonstrated more efficient tissue regeneration compared to using only a scaffold or cell-seeding therapy [167]. Additionally, decellularized grafts were found to provide proper reconstruction of rats’ small intestine, with a well-organized structure and intact nervous system in vivo [168]. Studies have shown that dECM scaffolds can create a favourable microenvironment that promotes tissue regeneration. They provide a tissue-specific template for the healing and functional regeneration of various tissues, such as skin [169], cartilage [160], or dense regular connective tissue [170]. During tissue regeneration, fibroblasts and other surrounding cells recreate the tissue environment. It would be advantageous to degrade the used material, making space for newly formed tissue and avoiding the need for surgical removal.

The future of biomimetic scaffolds involves the development of nanobiotechnology or nanotechnology to assemble and control the function of proteins [171]. Molecular biomimetics provides solutions for the control and fabrication of large-scale nanostructures to assemble materials in two and three dimensions. The concept of forming 4D scaffolds relies on adding the dimension of time in 3D biomaterial processing. Under the influence of external factors, such as heat or moisture, 4D biomaterials change their properties and shape in accordance with the controlled treatment process. Bioinspired 4D objects with multiple activities have been introduced as a new approach to producing highly complex smart materials [172]. In drug delivery, small-sized lipid nanoparticles (LNPs) have potential, but their poor stability and intracellular trafficking weaken their effectiveness. The potential of creating hydrophobic scaffolds can be increased by using transformed, self-assembled LNP containing lipids and proteins that have been previously used for gene or drug delivery [173]. The choice of scaffold depends on the specific biological application and intended usage, which can range from biotechnology to regenerative medicine for organ regeneration.

## 6. Application of Biomimetic Scaffolds in Biotechnology

Stem cells are a fundamental tool for tissue engineering and regenerative medicine due to their pluripotency and differentiation ability. The potential of stem cells is limited by their source, with the fertilized oocyte being the cell with the widest potency, giving rise to cells from all germ layers. Embryonic stem cells (ESCs) and induced pluripotent stem cells (iPSCs) are used in tissue engineering and are influenced by their environment [153]. ESCs are more effective than adult stem cells (ASCs) and can generate any organ through tissue-specific differentiation conditions in vitro. However, ASCs and tissue-specific cells are limited in number, making it difficult to grow a complete organ. The primary sources of stem cells for tissue engineering are bone marrow, umbilical cord blood, and circulating blood [174,175]. Recent stem cell research has shown the potential for nuclear transfer of somatic cells and reprogramming, which can convert fully or partially differentiated cells back to their embryonic state, restoring their stemness [176]. This technology has the advantage of using autologous cells, which helps to overcome issues with immune rejection. The extracellular matrix (ECM) plays a crucial role in determining stem cell fate. The chemical composition, surface chemistry, porosity, degradation behavior, and mechanical strength of a scaffold can all influence the fate of stem cells [177]. Both natural and synthetic biomaterials can create bioactive scaffolds that regulate stem cell differentiation [178]. Another biotool used in biotechnology is gene transfer, which involves managing RNA, DNA, proteins, and other macromolecules, such as lipids, for various purposes. There are different techniques of gene delivery for different biotechnological purposes, including viral and nonviral techniques. Nonviral techniques, such as transfection, can be performed through methods such as injection of naked DNA, electroporation, particle bombardment, and cationic liposomes. Contemporary viral gene transfer occurs in vivo using retroviruses, adenoviruses, or adeno-associated viruses. An alternative strategy involves incorporating DNA directly into a polymeric scaffold or genetically manipulated cells. This technique can protect DNA from degradation, offer better control of transgene and protein levels, and potentially reduce inflammation. In tissue engineering, transfections are utilized to deliver drugs or produce growth factors that can reduce the need for recombinant proteins, thus supporting tissue regeneration [179]. Scaffolds provide a more efficient method for gene delivery to specific cell populations while protecting vectors against extracellular barriers that may reduce their therapeutic efficacy and immune responses. Scaffolds can enhance cell adhesion, maintain cell–cell interaction, and increase protein expression levels [180]. They also offer the potential to alter cell function and fate. Hydrophobic scaffolds have been utilized for drug and gene delivery to treat diseases and develop DNA vaccines due to their increased adsorption capacity for proteins and other supplements [181]. Biopolymers can be used as a carrier for DNA vaccines at the organism scale. Plasmid DNA can be incorporated or surface-adsorbed onto carriers to create a complex that can be encapsulated in layers of other polymers or incorporated into multistructured polymer forms or matrices [182].

## 7. Application of Biomimetic Scaffolds in Advanced Regenerative and Reconstructive Medicine

For decades, scientists have recognized that the human body provides biological scaffolds and biopolymers that are highly attractive for tissue engineering and regenerative medicine. The human body has the ability to regenerate most organs, so damage to major organs can cause failure and serious disorders. Regenerative medicine combines tissue engineering with cell-based therapy, gene therapy, and immunomodulation. One potential use of dCEM scaffolds is for organ transplantation and tissue repair to regenerate and restore organ function [158]. Primary or genetically engineered cells can be transplanted by seeding them onto tissue-like three-dimensional structures to repair tissue damage. The traditional method of injecting cell solutions into tissues by hand encounters many difficulties, including high mortality rates for implanted cells. Biomimetic scaffolds consist of extracellular matrix (ECM) proteins and growth factors [148], which facilitate the transportation of high-density cells to affected tissues. Tissue engineering, a subfield of regenerative medicine, has the potential to regenerate almost all tissues and organs in the human body. Tissue engineering strategies generally involve implanting a construct into an organism, and delivering growth factors or supplements to enhance cell viability, proliferation, and adhesion to surrounding tissues [160]. Biomimetic materials are often used to replace unhealthy tissue in the body, while sutures are necessary for certain types of wounds. Fabrication methods can improve the production of customizable defect-fillable scaffolds for tissue regeneration in regenerative medicine [130]. Decellularized tissue can effectively replace native tissues as a biomimicking wound dressing [166]. Additionally, sutures for wound sewing can be manufactured with lyophilized decellularized ligament to biomimic the surrounding tissues. The spinning of fibers enabled the creation of thread that is as durable as silk fibers, which has potential use in wound sewing [105]. Biomimetic scaffolds have numerous applications in medicine, including artificial skin, arteries, and joint replacements in the body [183,184].

## 8. Conclusions and Future Perspectives

Considering the current state of knowledge regarding biomimetic scaffolds, it is difficult to overestimate their potential future applications. They have enhanced traditional 3D cell culture techniques by adding another dimension of modifiable parameters to already complex structures. Further advancement in the field of biomimetic scaffolds may be the branch of tissue engineering that will bring to light concepts like tailor-made organ implants that eliminate the risk of transplant rejection complications, laboratory modelling of patient-specific tumors, and the cultivation of healthy cells in an environment that closely mimics natural conditions. The potential applications of biomimetic scaffolds in regenerative, translational, and personalized medicine are potentially very vast, but due to high complexity of the constructs further extensive studies are still required.

## Data Availability

No new data were created or analyzed in this study.

## References

[1] Bertsch C., Maréchal H., Gribova V., Lévy B., Debry C., Lavalle P., Fath L. (2023). Biomimetic Bilayered Scaffolds for Tissue Engineering: From Current Design Strategies to Medical Applications. Adv. Health Mater..

[2] Naik R.R., Singamaneni S. (2017). Introduction: Bioinspired and Biomimetic Materials. Chem. Rev..

[3] Badekila A.K., Kini S., Jaiswal A.K. (2021). Fabrication techniques of biomimetic scaffolds in three-dimensional cell culture: A review. J. Cell. Physiol..

[4] Huang G., Li F., Zhao X., Ma Y., Li Y., Lin M., Jin G., Lu T.J., Genin G.M., Xu F. (2017). Functional and Biomimetic Materials for Engineering of the Three-Dimensional Cell Microenvironment. Chem. Rev..

[5] Zhang Y., Zhang C., Li Y., Zhou L., Dan N., Min J., Chen Y., Wang Y. (2023). Evolution of biomimetic ECM scaffolds from decellularized tissue matrix for tissue engineering: A comprehensive review. Int. J. Biol. Macromol..

[6] Vinatier C., Guicheux J., Daculsi G., Layrolle P., Weiss P. (2006). Cartilage and bone tissue engineering using hydrogels. Biomed. Mater. Eng..

[7] Flégeau K., Pace R., Gautier H., Rethore G., Guicheux J., Le Visage C., Weiss P. (2017). Toward the development of biomimetic injectable and macroporous biohydrogels for regenerative medicine. Adv. Colloid Interface Sci..

[8] Shin H., Jo S., Mikos A.G. (2003). Biomimetic materials for tissue engineering. Biomaterials.

[9] Mridha A.R., Dargaville T.R., Dalton P.D., Carroll L., Morris M.B., Vaithilingam V., Tuch B.E. (2022). Prevascularized Retrievable Hybrid Implant to Enhance Function of Subcutaneous Encapsulated Islets. Tissue Eng. Part A.

[10] Ogundipe V.M., Plukker J.T., Links T.P., Coppes R.P. (2022). Thyroid Gland Organoids: Current Models and Insights for Application in Tissue Engineering. Tissue Eng. Part A.

[11] A Salg G., Poisel E., Neulinger-Munoz M., Gerhardus J., Cebulla D., Bludszuweit-Philipp C., Vieira V., Nickel F., Herr I., Blaeser A. (2022). Toward 3D-bioprinting of an endocrine pancreas: A building-block concept for bioartificial insulin-secreting tissue. J. Tissue Eng..

[12] Gholami K., Solhjoo S., Aghamir S.M.K. (2022). Application of Tissue-Specific Extracellular Matrix in Tissue Engineering: Focus on Male Fertility Preservation. Reprod. Sci..

[13] Basurto I.M., Passipieri J.A., Gardner G.M., Smith K.K., Amacher A.R., Hansrisuk A.I., Christ G.J., Caliari S.R. (2022). Photoreactive Hydrogel Stiffness Influences Volumetric Muscle Loss Repair. Tissue Eng. Part A.

[14] Takahashi H., Oikawa F., Takeda N., Shimizu T. (2022). Contraction Control of Aligned Myofiber Sheet Tissue by Parallel Oriented Induced Pluripotent Stem Cell-Derived Neurons. Tissue Eng. Part A.

[15] Liu X., Zhang F., Li L., He Y., Dong Y. (2022). Reconstruction of Epidural Fat to Prevent Epidural Fibrosis After Laminectomy in Rabbits. Tissue Eng. Part A.

[16] Uemoto Y., Taura K., Nakamura D., Xuefeng L., Nam N.H., Kimura Y., Yoshino K., Fuji H., Yoh T., Nishio T. (2022). Bile Duct Regeneration with an Artificial Bile Duct Made of Gelatin Hydrogel Nonwoven Fabrics. Tissue Eng. Part A.

[17] Xiang Y., Wang W., Gao Y., Zhang J., Zhang J., Bai Z., Zhang S., Yang Y. (2020). Production and Characterization of an Integrated Multi-Layer 3D Printed PLGA/GelMA Scaffold Aimed for Bile Duct Restoration and Detection. Front. Bioeng. Biotechnol..

[18] Knox C., Garcia K., Tran J., Wilson S.M., Blood A.B., Kearns-Jonker M., Martens T.P. (2023). A Biomimetic Approach Utilizing Pulsatile Perfusion Generates Contractile Vascular Grafts. Tissue Eng. Part A.

[19] Szöőr Á., Ujlaky-Nagy L., Tóth G., Szöllősi J., Vereb G. (2016). Cell confluence induces switching from proliferation to migratory signaling by site-selective phosphorylation of PDGF receptors on lipid raft platforms. Cell. Signal..

[20] Hamburger A.W., Salmon S.E. (1977). Primary bioassay of human tumor stem cells. Science.

[21] Jensen C., Teng Y. (2020). Is It Time to Start Transitioning From 2D to 3D Cell Culture?. Front. Mol. Biosci..

[22] Langhans S.A. (2018). Three-Dimensional in Vitro Cell Culture Models in Drug Discovery and Drug Repositioning. Front. Pharmacol..

[23] Kapałczyńska M., Kolenda T., Przybyła W., Zajączkowska M., Teresiak A., Filas V., Ibbs M., Bliźniak R., Łuczewski Ł., Lamperska K. (2018). 2D and 3D cell cultures—A comparison of different types of cancer cell cultures. Arch. Med. Sci..

[24] Biju T.S., Priya V.V., Francis A.P. (2023). Role of three-dimensional cell culture in therapeutics and diagnostics: An updated review. Drug Deliv. Transl. Res..

[25] Abbott A. (2003). Biology’s new dimension. Nature.

[26] Habanjar O., Diab-Assaf M., Caldefie-Chezet F., Delort L. (2021). 3D Cell Culture Systems: Tumor Application, Advantages, and Disadvantages. Int. J. Mol. Sci..

[27] Anthon S.G., Valente K.P. (2022). Vascularization Strategies in 3D Cell Culture Models: From Scaffold-Free Models to 3D Bioprinting. Int. J. Mol. Sci..

[28] Sośniak J., Opiela J. (2021). 3D Cell Culture Technology—A New Insight Into Research—A Review. Ann. Anim. Sci..

[29] Fontoura J.C., Viezzer C., dos Santos F.G., Ligabue R.A., Weinlich R., Puga R.D., Antonow D., Severino P., Bonorino C. (2020). Comparison of 2D and 3D cell culture models for cell growth, gene expression and drug resistance. Mater. Sci. Eng. C Mater. Biol. Appl..

[30] Sakalem M.E., De Sibio M.T., da Costa F.A.d.S., de Oliveira M. (2021). Historical evolution of spheroids and organoids, and possibilities of use in life sciences and medicine. Biotechnol. J..

[31] Arjmand B., Rabbani Z., Soveyzi F., Tayanloo-Beik A., Rezaei-Tavirani M., Biglar M., Adibi H., Larijani B. (2023). Advancement of Organoid Technology in Regenerative Medicine. Regen. Eng. Transl. Med..

[32] Cui X., Hartanto Y., Zhang H. (2017). Advances in multicellular spheroids formation. J. R. Soc. Interface.

[33] Białkowska K., Komorowski P., Bryszewska M., Miłowska K. (2020). Spheroids as a Type of Three-Dimensional Cell Cultures—Examples of Methods of Preparation and the Most Important Application. Int. J. Mol. Sci..

[34] Liu D., Chen S., Naing M.W. (2021). A review of manufacturing capabilities of cell spheroid generation technologies and future development. Biotechnol. Bioeng..

[35] Sant S., Johnston P.A. (2017). The production of 3D tumor spheroids for cancer drug discovery. Drug Discov. Today Technol..

[36] Shyam R., Reddy L.V.K., Palaniappan A. (2023). Fabrication and Characterization Techniques of In Vitro 3D Tissue Models. Int. J. Mol. Sci..

[37] Madhavan M., Jaiswal D., Karlberg S., Duggan A., Almarshad H.A., Claffey K.P., Hoshino K. (2023). Electron microscopy imaging and mechanical characterization of T47D multicellular tumor spheroids–Older spheroids reduce interstitial space and become stiffer. PLoS ONE.

[38] Tosca E.M., Ronchi D., Facciolo D., Magni P. (2023). Replacement, Reduction, and Refinement of Animal Experiments in Anticancer Drug Development: The Contribution of 3D In Vitro Cancer Models in the Drug Efficacy Assessment. Biomedicines.

[39] Yau J.N.N., Adriani G. (2023). Three-dimensional heterotypic colorectal cancer spheroid models for evaluation of drug response. Front. Oncol..

[40] Zhang Y., Huo J., Yu S., Feng W., Tuersun A., Chen F., Lv Z., Liu W., Zhao J., Xu Z. (2023). Colorectal cancer tissue-originated spheroids reveal tumor intrinsic signaling pathways and mimic patient clinical chemotherapeutic response as a rapid and valid model. Biomed. Pharmacother..

[41] El-Sadek I.A., Shen L.T.-W., Mori T., Makita S., Mukherjee P., Lichtenegger A., Matsusaka S., Yasuno Y. (2023). Label-free drug response evaluation of human derived tumor spheroids using three-dimensional dynamic optical coherence tomography. Sci. Rep..

[42] Răileanu M., Bacalum M. (2023). Cancer Wars: Revenge of the AMPs (Antimicrobial Peptides), a New Strategy against Colorectal Cancer. Toxins.

[43] Domingues M., Pereira C.L., Sarmento B., Castro F. (2023). Mimicking 3D breast tumor-stromal interactions to screen novel cancer therapeutics. Eur. J. Pharm. Sci..

[44] Khan A.H., Zhou S.P., Moe M., Quesada B.A.O., Bajgiran K.R., Lassiter H.R., Dorman J.A., Martin E.C., Pojman J.A., Melvin A.T. (2022). Generation of 3D Spheroids Using a Thiol–Acrylate Hydrogel Scaffold to Study Endocrine Response in ER^+^ Breast Cancer. ACS Biomater. Sci. Eng..

[45] Klingseisen V., Slanovc J., Regouc M., Hrzenjak A. (2022). Bisdemethoxycurcumin sensitizes the response of cisplatin resistant non-small cell lung carcinoma cell lines by activating apoptosis and autophagy. J. Nutr. Biochem..

[46] Huang M., Hou W., Zhang J., Li M., Zhang Z., Li X., Chen Z., Wang C., Yang L. (2022). Evaluation of AMG510 Therapy on *KRAS*-Mutant Non–Small Cell Lung Cancer and Colorectal Cancer Cell Using a 3D Invasive Tumor Spheroid System under Normoxia and Hypoxia. Bioengineering.

[47] Świerczewska M., Sterzyńska K., Ruciński M., Andrzejewska M., Nowicki M., Januchowski R. (2023). The response and resistance to drugs in ovarian cancer cell lines in 2D monolayers and 3D spheroids. Biomed. Pharmacother..

[48] O’brien S., Butticello M., Thompson C., Wilson B., Wyce A., Mahajan V., Kruger R., Mohammad H., Fedoriw A. (2023). Inhibiting PRMT5 induces DNA damage and increases anti-proliferative activity of Niraparib, a PARP inhibitor, in models of breast and ovarian cancer. BMC Cancer.

[49] Fevre R., Mary G., Vertti-Quintero N., Durand A., Tomasi R.F.-X., Del Nery E., Baroud C.N. (2023). Combinatorial drug screening on 3D Ewing sarcoma spheroids using droplet-based microfluidics. iScience.

[50] Moskovits N., Itzhaki E., Tarasenko N., Chausky E., Bareket-Samish A., Kaufman A., Meerson R., Stemmer S.M. (2022). Establishing 3-Dimensional Spheroids from Patient-Derived Tumor Samples and Evaluating their Sensitivity to Drugs. J. Vis. Exp..

[51] Zhang W., Kyritsi K., Isidan A., Park Y., Li P., Cross-Najafi A.A., Lopez K., Kennedy L., Sato K., Glaser S. (2023). Development of Scaffold-Free Three-Dimensional Cholangiocyte Organoids to Study the Progression of Primary Sclerosing Cholangitis. Am. J. Pathol..

[52] Yang Q., Li M., Yang X., Xiao Z., Tong X., Tuerdi A., Li S., Lei L. (2023). Flourishing tumor organoids: History, emerging technology, and application. Bioeng. Transl. Med..

[53] Xu Z.-Y., Huang J.-J., Liu Y., Zhao Y., Wu X.-W., Ren J.-A. (2021). Current knowledge on the multiform reconstitution of intestinal stem cell niche. World J. Stem Cells.

[54] Kim H., Kamm R.D., Vunjak-Novakovic G., Wu J.C. (2022). Progress in multicellular human cardiac organoids for clinical applications. Cell Stem Cell.

[55] Li J., Chu J., Lui V.C.H., Chen S., Chen Y., Tam P.K.H. (2022). Bioengineering Liver Organoids for Diseases Modelling and Transplantation. Bioengineering.

[56] Ma X., Wang Q., Li G., Li H., Xu S., Pang D. (2023). Cancer organoids: A platform in basic and translational research. Genes Dis..

[57] Chan W.S., Mo X., Ip P.P.C., Tse K.Y. (2023). Patient-derived organoid culture in epithelial ovarian cancers—Techniques, applications, and future perspectives. Cancer Med..

[58] Li Y., Gao X., Ni C., Zhao B., Cheng X. (2023). The application of patient-derived organoid in the research of lung cancer. Cell. Oncol..

[59] Nizamoglu M., Joglekar M.M., Almeida C.R., Callerfelt A.-K.L., Dupin I., Guenat O.T., Henrot P., van Os L., Otero J., Elowsson L. (2023). Innovative three-dimensional models for understanding mechanisms underlying lung diseases: Powerful tools for translational research. Eur. Respir. Rev..

[60] Septiana W.L., Noviantari A., Antarianto R.D. (2023). Induced Pluripotent Stem Cells (Ipscs) Based Liver Organoid: The Benefits and Challenges. Cell. Physiol. Biochem..

[61] Wright C.W., Li N., Shaffer L., Hill A., Boyer N., Alves S.E., Venkataraman S., Biswas K., Lieberman L.A., Mohammadi S. (2023). Establishment of a 96-well transwell system using primary human gut organoids to capture multiple quantitative pathway readouts. Sci. Rep..

[62] Zhang X.P., Wang X.Y., Wang S.N., Miao C.Y. (2023). The generation and properties of human cortical organoids as a disease model for malformations of cortical development. Neural Regen. Res..

[63] Yan Z., Zhang T., Wang Y., Xiao S., Gao J. (2023). Extracellular vesicle biopotentiated hydrogels for diabetic wound healing: The art of living nanomaterials combined with soft scaffolds. Mater. Today Bio.

[64] Gan Z., Qin X., Liu H., Liu J., Qin J. (2023). Recent advances in defined hydrogels in organoid research. Bioact. Mater..

[65] Willemse J., van der Laan L.J.W., de Jonge J., Verstegen M.M.A. (2022). Design by Nature: Emerging Applications of Native Liver Extracellular Matrix for Cholangiocyte Organoid-Based Regenerative Medicine. Bioengineering.

[66] Ho T.-C., Chang C.-C., Chan H.-P., Chung T.-W., Shu C.-W., Chuang K.-P., Duh T.-H., Yang M.-H., Tyan Y.-C. (2022). Hydrogels: Properties and Applications in Biomedicine. Molecules.

[67] Deptuła M., Zawrzykraj M., Sawicka J., Banach-Kopeć A., Tylingo R., Pikuła M. (2023). Application of 3D- printed hydrogels in wound healing and regenerative medicine. Biomed. Pharmacother..

[68] Kane K.I.W., Moreno E.L., Lehr C.M., Hachi S., Dannert R., Sanctuary R., Wagner C., Fleming R.M.T., Baller J. (2018). Determination of the rheological properties of Matrigel for optimum seeding conditions in microfluidic cell cultures. AIP Adv..

[69] Tang C., Zhou K., Zhu Y., Zhang W., Xie Y., Wang Z., Zhou H., Yang T., Zhang Q., Xu B. (2022). Collagen and its derivatives: From structure and properties to their applications in food industry. Food Hydrocoll..

[70] Salahuddin B., Wang S., Sangian D., Aziz S., Gu Q. (2021). Hybrid Gelatin Hydrogels in Nanomedicine Applications. ACS Appl. Bio Mater..

[71] Saravanakumar K., Park S., Santosh S.S., Ganeshalingam A., Thiripuranathar G., Sathiyaseelan A., Vijayasarathy S., Swaminathan A., Priya V.V., Wang M.-H. (2022). Application of hyaluronic acid in tissue engineering, regenerative medicine, and nanomedicine: A review. Int. J. Biol. Macromol..

[72] Roquero D.M., Katz E. (2022). “Smart” alginate hydrogels in biosensing, bioactuation and biocomputing: State-of-the-art and perspectives. Sensors Actuators Rep..

[73] Ashammakhi N., GhavamiNejad A., Tutar R., Fricker A., Roy I., Chatzistavrou X., Apu E.H., Nguyen K.-L., Ahsan T., Pountos I. (2022). Highlights on Advancing Frontiers in Tissue Engineering. Tissue Eng. Part B Rev..

[74] Banerjee D., Singh Y.P., Datta P., Ozbolat V., O’Donnell A., Yeo M., Ozbolat I.T. (2022). Strategies for 3D bioprinting of spheroids: A comprehensive review. Biomaterials.

[75] Jin R.H., Zhang Z.Z., Xu P.Q., Xia S.Z., Weng T.T., Zhu Z.K., Wang X.G., You C.G., Han C.M. (2023). Effects of three-dimensional bioprinting antibacterial hydrogel on full-thickness skin defect wounds in rats. Zhonghua Shao Shang Yu Chuang Mian Xiu Fu Za Zhi.

[76] Agarwal N., Solanki V.S., Ameta K.L., Yadav V.K., Gupta P., Wanale S.G., Shrivastava R., Soni A., Sahoo D.K., Patel A. (2023). 4-Dimensional printing: Exploring current and future capabilities in biomedical and healthcare systems—A Concise review. Front. Bioeng. Biotechnol..

[77] Vyas J., Shah I., Singh S., Prajapati B.G. (2023). Biomaterials-based additive manufacturing for customized bioengineering in management of otolaryngology: A comprehensive review. Front. Bioeng. Biotechnol..

[78] Trenfield S.J., Awad A., Madla C.M., Hatton G.B., Firth J., Goyanes A., Gaisford S., Basit A.W. (2019). Shaping the future: Recent advances of 3D printing in drug delivery and healthcare. Expert Opin. Drug Deliv..

[79] Li H., Go G., Ko S.Y., Park J.-O., Park S. (2016). Magnetic actuated pH-responsive hydrogel-based soft micro-robot for targeted drug delivery. Smart Mater. Struct..

[80] Ying B., Liu X. (2021). Skin-like hydrogel devices for wearable sensing, soft robotics and beyond. iScience.

[81] Chen A., Wang W., Mao Z., He Y., Chen S., Liu G., Su J., Feng P., Shi Y., Yan C. (2023). Multimaterial 3D and 4D Bioprinting of Heterogenous Constructs for Tissue Engineering. Adv. Mater..

[82] Loverde J.R., Piroli M., Klarmann G.J., Gaston J., Wickiser J.K., Barnhill J., Gilchrist K.H., Ho V.B. (2023). Development of a bioreactor for in-vitro compression cycling of tissue engineered meniscal implants. HardwareX.

[83] Agarwal T., Chiesa I., Costantini M., Lopamarda A., Tirelli M.C., Borra O.P., Varshapally S.V.S., Kumar Y.A.V., Reddy G.K., De Maria C. (2023). Chitosan and its derivatives in 3D/4D (bio) printing for tissue engineering and drug delivery applications. Int. J. Biol. Macromol..

[84] Zhang X., Yang Y., Yang Z., Ma R., Aimaijiang M., Xu J., Zhang Y., Zhou Y. (2023). Four-Dimensional Printing and Shape Memory Materials in Bone Tissue Engineering. Int. J. Mol. Sci..

[85] Kang X., Zhang X.-B., Gao X.-D., Hao D.-J., Li T., Xu Z.-W. (2022). Bioprinting for bone tissue engineering. Front. Bioeng. Biotechnol..

[86] Gokdogan O. (2022). Bone grafting in maxillofacial trauma. Curr. Opin. Otolaryngol. Head Neck Surg..

[87] Yazdanpanah Z., Johnston J.D., Cooper D.M.L., Chen X. (2022). 3D Bioprinted Scaffolds for Bone Tissue Engineering: State-Of-The-Art and Emerging Technologies. Front. Bioeng. Biotechnol..

[88] Chen X., Han S., Wu W., Wu Z., Yuan Y., Wu J., Liu C. (2022). Harnessing 4D Printing Bioscaffolds for Advanced Orthopedics. Small.

[89] Kabirian F., Mela P., Heying R. (2022). 4D Printing Applications in the Development of Smart Cardiovascular Implants. Front. Bioeng. Biotechnol..

[90] Aizarna-Lopetegui U., García-Astrain C., Renero-Lecuna C., González-Callejo P., Villaluenga I., del Pozo M.A., Sánchez-Álvarez M., Henriksen-Lacey M., de Aberasturi D.J. (2023). Remodeling arteries: Studying the mechanical properties of 3D-bioprinted hybrid photoresponsive materials. J. Mater. Chem. B.

[91] Zhou Z., Tang W., Yang J., Fan C. (2023). Application of 4D printing and bioprinting in cardiovascular tissue engineering. Biomater. Sci..

[92] Zieliński P.S., Gudeti P.K.R., Rikmanspoel T., Włodarczyk-Biegun M.K. (2022). 3D printing of bio-instructive materials: Toward directing the cell. Bioact. Mater..

[93] Deshpande M.V., West A.J., Bernacki S.H., Luan K., King M.W. (2020). Poly(ε-Caprolactone) Resorbable Auxetic Designed Knitted Scaffolds for Craniofacial Skeletal Muscle Regeneration. Bioengineering.

[94] Wang Z., Liang X., Wang G., Wang X., Chen Y. (2023). Emerging Bioprinting for Wound Healing. Adv. Mater..

[95] Borse K., Shende P. (2023). 3D-to-4D Structures: An Exploration in Biomedical Applications. Aaps Pharmscitech.

[96] Douillet C., Nicodeme M., Hermant L., Bergeron V., Guillemot F., Fricain J.-C., Oliveira H., Garcia M. (2022). From local to global matrix organization by fibroblasts: A 4D laser-assisted bioprinting approach. Biofabrication.

[97] Xie N., Shi G., Shen Y., Xu Y., Wang H., Feng H., Dai K., Wang J., Cao Q. (2022). Research Progress of Robot Technology in In situ 3D Bioprinting. Int. J. Bioprinting.

[98] Jongprasitkul H., Parihar V.S., Turunen S., Kellomäki M. (2023). pH-Responsive Gallol-Functionalized Hyaluronic Acid-Based Tissue Adhesive Hydrogels for Injection and Three-Dimensional Bioprinting. ACS Appl. Mater. Interfaces.

[99] Zhou B., Jiang X., Zhou X., Tan W., Luo H., Lei S., Yang Y. (2023). GelMA-based bioactive hydrogel scaffolds with multiple bone defect repair functions: Therapeutic strategies and recent advances. Biomater. Res..

[100] Bhattacharyya A., Ham H.-W., Sonh J., Gunbayar M., Jeffy R., Nagarajan R., Khatun M.R., Noh I. (2023). 3D bioprinting of complex tissue scaffolds with in situ homogeneously mixed alginate-chitosan-kaolin bioink using advanced portable biopen. Carbohydr. Polym..

[101] Fani N., Peshkova M., Bikmulina P., Golroo R., Timashev P., Vosough M. (2023). Fabricating the cartilage: Recent achievements. Cytotechnology.

[102] Kiyotake E.A., Thomas E.E., Iribagiza C., Detamore M.S. (2023). High-stiffness, fast-crosslinking, cartilage matrix bioinks. J. Biomech..

[103] Hafeez S., Decarli M.C., Aldana A., Ebrahimi M., Ruiter F.A., Duimel H., van Blitterswijk C., Pitet L.M., Moroni L., Baker M.B. (2023). In Situ Covalent Reinforcement of a Benzene-1,3,5-Tricarboxamide Supramolecular Polymer Enables Biomimetic, Tough, and Fibrous Hydrogels and Bioinks. Adv. Mater..

[104] Yang X., Li X., Wu Z., Cao L. (2023). Photocrosslinked methacrylated natural macromolecular hydrogels for tissue engineering: A review. Int. J. Biol. Macromol..

[105] Zhou F., Xin L., Wang S., Chen K., Li D., Wang S., Huang Y., Xu C., Zhou M., Zhong W. (2023). Portable Handheld “SkinPen” Loaded with Biomaterial Ink for In Situ Wound Healing. ACS Appl. Mater. Interfaces.

[106] Chen H., Ma X., Gao T., Zhao W., Xu T., Liu Z. (2023). Robot-assisted in situ bioprinting of gelatin methacrylate hydrogels with stem cells induces hair follicle-inclusive skin regeneration. Biomed. Pharmacother..

[107] Zhang F., Zhang Z., Duan X., Song W., Li Z., Yao B., Kong Y., Huang X., Fu X., Chang J. (2023). Integrating zinc/silicon dual ions with 3D-printed GelMA hydrogel promotes in situ hair follicle regeneration. Int. J. Bioprinting.

[108] Fortunato G.M., Sigismondi S., Nicoletta M., Condino S., Montemurro N., Vozzi G., Ferrari V., De Maria C. (2023). Analysis of the Robotic-Based In Situ Bioprinting Workflow for the Regeneration of Damaged Tissues through a Case Study. Bioengineering.

[109] Yang Y., Yu Z., Lu X., Dai J., Zhou C., Yan J., Wang L., Wang Z., Zang J. (2023). Minimally invasive bioprinting for in situ liver regeneration. Bioact. Mater..

[110] Thai M.T., Phan P.T., Tran H.A., Nguyen C.C., Hoang T.T., Davies J., Rnjak-Kovacina J., Phan H., Lovell N.H., Do T.N. (2023). Advanced Soft Robotic System for In Situ 3D Bioprinting and Endoscopic Surgery. Adv. Sci..

[111] Zhang S., Qi C., Zhang W., Zhou H., Wu N., Yang M., Meng S., Liu Z., Kong T. (2023). In Situ Endothelialization of Free-Form 3D Network of Interconnected Tubular Channels via Interfacial Coacervation by Aqueous-in-Aqueous Embedded Bioprinting. Adv. Mater..

[112] Dong H., Hu B., Zhang W., Xie W., Mo J., Sun H., Shang J. (2022). Robotic-assisted automated in situ bioprinting. Int. J. Bioprinting.

[113] Li T., Hou J., Wang L., Zeng G., Wang Z., Yu L., Yang Q., Yin J., Long M., Chen L. (2023). Bioprinted anisotropic scaffolds with fast stress relaxation bioink for engineering 3D skeletal muscle and repairing volumetric muscle loss. Acta Biomater..

[114] Moncal K.K., Yeo M., Celik N., Acri T.M., Rizk E., Wee H., Lewis G.S., Salem A.K., Ozbolat I.T. (2022). Comparison of in-situ versus ex-situ delivery of polyethylenimine-BMP-2 polyplexes for rat calvarial defect repair via intraoperative bioprinting. Biofabrication.

[115] Shen M., Wang L., Gao Y., Feng L., Xu C., Li S., Wang X., Wu Y., Guo Y., Pei G. (2022). 3D bioprinting of in situ vascularized tissue engineered bone for repairing large segmental bone defects. Mater. Today Bio.

[116] Huo Y., Xu Y., Wu X., Gao E., Zhan A., Chen Y., Zhang Y., Hua Y., Swieszkowski W., Zhang Y.S. (2022). Functional Trachea Reconstruction Using 3D-Bioprinted Native-Like Tissue Architecture Based on Designable Tissue-Specific Bioinks. Adv. Sci..

[117] Campos D.F.D., Zhang S., Kreimendahl F., Köpf M., Fischer H., Vogt M., Blaeser A., Apel C., Esteves-Oliveira M. (2020). Hand-held bioprinting for de novo vascular formation applicable to dental pulp regeneration. Connect. Tissue Res..

[118] Farto-Vaamonde X., Diaz-Gomez L., Parga A., Otero A., Concheiro A., Alvarez-Lorenzo C. (2022). Perimeter and carvacrol-loading regulate angiogenesis and biofilm growth in 3D printed PLA scaffolds. J. Control. Release.

[119] Zarei M., Sayedain S.S., Askarinya A., Sabbaghi M., Alizadeh R. (2023). Improving physio-mechanical and biological properties of 3D-printed PLA scaffolds via in-situ argon cold plasma treatment. Sci. Rep..

[120] Kim S.J., Lee G., Park J.-K. (2023). Hybrid Biofabrication of Heterogeneous 3D Constructs Using Low-Viscosity Bioinks. ACS Appl. Mater. Interfaces.

[121] Custodio C.L., Broñola P.J.M., Cayabyab S.R., Lagura V.U., Celorico J.R., Basilia B.A. (2021). Powder Loading Effects on the Physicochemical and Mechanical Properties of 3D Printed Poly Lactic Acid/Hydroxyapatite Biocomposites. Int. J. Bioprint..

[122] Fan G., Yang L., Liu D., Wang Y., Ji W., Tukebai, Qin H., Wang Z. (2022). Repair of Cranial Defects in Rabbits with 3D-Printed Hydroxyapatite/Polylactic Acid Composites. BioMed Res. Int..

[123] Sultan S., Thomas N., Varghese M., Dalvi Y., Joy S., Hall S., Mathew A.P. (2022). The Design of 3D-Printed Polylactic Acid–Bioglass Composite Scaffold: A Potential Implant Material for Bone Tissue Engineering. Molecules.

[124] Cichos S., Schätzlein E., Wiesmann-Imilowski N., Blaeser A., Henrich D., Frank J., Drees P., Gercek E., Ritz U. (2023). A new 3D-printed polylactic acid-bioglass composite for bone tissue engineering induces angiogenesis in vitro and in ovo. Int. J. Bioprinting.

[125] Guo F., Wang E., Yang Y., Mao Y., Liu C., Bu W., Li P., Zhao L., Jin Q., Liu B. (2023). A natural biomineral for enhancing the biomineralization and cell response of 3D printed polylactic acid bone scaffolds. Int. J. Biol. Macromol..

[126] Jang H.J., Kang M.S., Kim W.-H., Jo H.J., Lee S.-H., Hahm E.J., Oh J.H., Hong S.W., Kim B., Han D.-W. (2023). 3D printed membranes of polylactic acid and graphene oxide for guided bone regeneration. Nanoscale Adv..

[127] Pisani S., Mauri V., Negrello E., Mauramati S., Alaimo G., Auricchio F., Benazzo M., Dorati R., Genta I., Conti B. (2023). Assessment of different manufacturing techniques for the production of bioartificial scaffolds as soft organ transplant substitutes. Front. Bioeng. Biotechnol..

[128] Duan R., Wang Y., Zhang Y., Wang Z., Du F., Du B., Su D., Liu L., Li X., Zhang Q. (2021). Blending with Poly(l-lactic acid) Improves the Printability of Poly(l-lactide-*co*-caprolactone) and Enhances the Potential Application in Cartilage Tissue Engineering. ACS Omega.

[129] Kim S.H., Kim D., Cha M., Kim S.H., Jung Y. (2021). The Regeneration of Large-Sized and Vascularized Adipose Tissue Using a Tailored Elastic Scaffold and dECM Hydrogels. Int. J. Mol. Sci..

[130] Lee S., Lee H.S., Chung J.J., Kim S.H., Park J.W., Lee K., Jung Y. (2021). Enhanced Regeneration of Vascularized Adipose Tissue with Dual 3D-Printed Elastic Polymer/dECM Hydrogel Complex. Int. J. Mol. Sci..

[131] Yin H., Zhu M., Wang Y., Luo L., Ye Q., Lee B.H. (2023). Physical properties and cellular responses of gelatin methacryloyl bulk hydrogels and highly ordered porous hydrogels. Front. Soft Matter.

[132] Choi J.R., Yong K.W., Choi J.Y., Cowie A.C. (2019). Recent advances in photo-crosslinkable hydrogels for biomedical applications. BioTechniques.

[133] Liang J., Wang Z., Poot A.A., Grijpma D.W., Dijkstra P.J., Wang R. (2023). Enzymatic post-crosslinking of printed hydrogels of methacrylated gelatin and tyramine-conjugated 8-arm poly(ethylene glycol) to prepare interpenetrating 3D network structures. Int. J. Bioprint..

[134] Wu W., Dong Y., Liu H., Jiang X., Yang L., Luo J., Hu Y., Gou M. (2023). 3D printed elastic hydrogel conduits with 7,8-dihydroxyflavone release for peripheral nerve repair. Mater. Today Bio.

[135] Ling Z., Zhao J., Song S., Xiao S., Wang P., An Z., Fu Z., Shao J., Zhang Z., Fu W. (2023). Chitin nanocrystal-assisted 3D bioprinting of gelatin methacrylate scaffolds. Regen. Biomater..

[136] Xu Z., Qi X., Bao M., Zhou T., Shi J., Xu Z., Zhou M., Boccaccini A.R., Zheng K., Jiang X. (2023). Biomineralization inspired 3D printed bioactive glass nanocomposite scaffolds orchestrate diabetic bone regeneration by remodeling micromilieu. Bioact. Mater..

[137] Pérez-Cortez J.E., Sánchez-Rodríguez V.H., Gallegos-Martínez S., Chuck-Hernández C., Rodriguez C.A., Álvarez M.M., Santiago G.T.-D., Vázquez-Lepe E., Martínez-López J.I. (2022). Low-Cost Light-Based GelMA 3D Bioprinting via Retrofitting: Manufacturability Test and Cell Culture Assessment. Micromachines.

[138] Elomaa L., Keshi E., Sauer I.M., Weinhart M. (2020). Development of GelMA/PCL and dECM/PCL resins for 3D printing of acellular in vitro tissue scaffolds by stereolithography. Mater. Sci. Eng. C Mater. Biol. Appl..

[139] Kumar H., Sakthivel K., Mohamed M.G.A., Boras E., Shin S.R., Kim K. (2021). Designing Gelatin Methacryloyl (GelMA)-Based Bioinks for Visible Light Stereolithographic 3D Biofabrication. Macromol. Biosci..

[140] Jurczyk M., Kasperczyk J., Wrześniok D., Beberok A., Jelonek K. (2022). Nanoparticles Loaded with Docetaxel and Resveratrol as an Advanced Tool for Cancer Therapy. Biomedicines.

[141] Sankaralingam P., Sakthivel P., Thangavel V.C., Sankaralingam P., Sakthivel P., Thangavel V.C. (2022). Novel Composites for Bone Tissue Engineering. Biomimetics—Bridging the Gap.

[142] Guerra A.J., Lammel-Lindemann J., Katko A., Kleinfehn A., Rodriguez C.A., Catalani L.H., Becker M.L., Ciurana J., Dean D. (2019). Optimization of photocrosslinkable resin components and 3D printing process parameters. Acta Biomater..

[143] Kondiah P.J., Kondiah P.P.D., Choonara Y.E., Marimuthu T., Pillay V. (2020). A 3D Bioprinted Pseudo-Bone Drug Delivery Scaffold for Bone Tissue Engineering. Pharmaceutics.

[144] Kolan K.C.R., Huang Y.-W., Semon J.A., Leu M.C. (2020). 3D-printed Biomimetic Bioactive Glass Scaffolds for Bone Regeneration in Rat Calvarial Defects. Int. J. Bioprint..

[145] Cheng K.-C., Sun Y.-M., Hsu S.-H. (2023). Development of double network polyurethane–chitosan composite bioinks for soft neural tissue engineering. J. Mater. Chem. B.

[146] Sawant M., Wang F., Koester J., Niehoff A., Nava M.M., Lundgren-Akerlund E., Gullberg D., Leitinger B., Wickström S., Eckes B. (2023). Ablation of integrin-mediated cell–collagen communication alleviates fibrosis. Ann. Rheum. Dis..

[147] Ahn S., Sharma U., Kasuba K.C., Strohmeyer N., Müller D.J. (2023). Engineered Biomimetic Fibrillar Fibronectin Matrices Regulate Cell Adhesion Initiation, Migration, and Proliferation via *α*5*β*1 Integrin and Syndecan-4 Crosstalk. Adv. Sci..

[148] Tiwari S., Patil R., Bahadur P. (2018). Polysaccharide Based Scaffolds for Soft Tissue Engineering Applications. Polymers.

[149] Jung S.A., Malyaran H., Demco D.E., Manukanc A., Häser L.S., Kučikas V., van Zandvoort M., Neuss S., Pich A. (2023). Fibrin–Dextran Hydrogels with Tunable Porosity and Mechanical Properties. Biomacromolecules.

[150] Ma C., Wang T., Jin X., Zhang W., Lv Q. (2023). Lineage-specific multifunctional double-layer scaffold accelerates the integrated regeneration of cartilage and subchondral bone. Mater. Today Bio.

[151] Ozturk T., Erpala F., Bozduman O., Gedikbas M., Eren M.B., Zengin E.C. (2023). Arthroscopic Treatment of Femoral Condyle Chondral Lesions: Microfracture Versus Liquid Bioscaffold. Indian J. Orthop..

[152] Zineh B.R., Roshangar L., Meshgi S., Shabgard M. (2022). 3D printing of alginate/thymoquinone/halloysite nanotube bio-scaffolds for cartilage repairs: Experimental and numerical study. Med. Biol. Eng. Comput..

[153] Patel D.K., Dutta S.D., Lim K.-T. (2019). Nanocellulose-based polymer hybrids and their emerging applications in biomedical engineering and water purification. RSC Adv..

[154] Konya P., Konya M.N., Yilmaz B.K., Kaga E., Kaga S., Çetinkol Y. (2023). Comparison of the Therapeutic Efficacy of Antibiotic-Loaded Polymeric Tissue Scaffold and Bone Cement in the Regeneration of Infected Bone Tissue. Cureus.

[155] Štiglic A.D., Gürer F., Lackner F., Bračič D., Winter A., Gradišnik L., Makuc D., Kargl R., Duarte I., Plavec J. (2022). Organic acid cross-linked 3D printed cellulose nanocomposite bioscaffolds with controlled porosity, mechanical strength, and biocompatibility. iScience.

[156] Aeberhard P., Grognuz A., Peneveyre C., McCallin S., Hirt-Burri N., Antons J., Pioletti D., Raffoul W., Applegate L.A. (2020). Efficient decellularization of equine tendon with preserved biomechanical properties and cytocompatibility for human tendon surgery indications. Artif. Organs.

[157] Tan Y., Fan S., Wu X., Liu M., Dai T., Liu C., Ni S., Wang J., Yuan X., Zhao H. (2023). Fabrication of a three-dimensional printed gelatin/sodium alginate/nano-attapulgite composite polymer scaffold loaded with leonurine hydrochloride and its effects on osteogenesis and vascularization. Int. J. Biol. Macromol..

[158] Jin J., Chen Z., Xiang Y., Tang T., Zhou H., Hong X., Fan H., Zhang X., Luo P., Ma B. (2020). Development of a PHMB hydrogel-modified wound scaffold dressing with antibacterial activity. Wound Repair Regen..

[159] Trapani G., Weiß M.S., Trappmann B. (2023). Tunable Synthetic Hydrogels to Study Angiogenic Sprouting. Curr. Protoc..

[160] Wei Q., Liu D., Chu G., Yu Q., Liu Z., Li J., Meng Q., Wang W., Han F., Li B. (2022). TGF-β1-supplemented decellularized annulus fibrosus matrix hydrogels promote annulus fibrosus repair. Bioact. Mater..

[161] Matsubayashi Y., Sánchez-Sánchez B.J., Marcotti S., Serna-Morales E., Dragu A., Díaz-De-La-Loza M.-D., Vizcay-Barrena G., Fleck R.A., Stramer B.M., Matsubayashi Y. (2020). Rapid Homeostatic Turnover of Embryonic ECM during Tissue Morphogenesis. Dev. Cell.

[162] Lee S.-Y., Robertson C., Diot A., Meuray V., Bourdon J.-C., Bissell M.J. (2022). Δ133p53 coordinates ECM-driven morphogenesis and gene expression in three-dimensional mammary epithelial acini. J. Cell Sci..

[163] Ma M., Hua S., Min X., Wang L., Li J., Wu P., Liang H., Zhang B., Chen X., Xiang S. (2022). p53 positively regulates the proliferation of hepatic progenitor cells promoted by laminin-521. Signal Transduct. Target. Ther..

[164] Furuta S., Ren G., Mao J.-H., Bissell M.J. (2018). Laminin signals initiate the reciprocal loop that informs breast-specific gene expression and homeostasis by activating NO, p53 and microRNAs. eLife.

[165] Rederer A., Rose V., Krüger R., Schmittutz L., Swierzy I., Fischer L., Thievessen I., Bauer J., Friedrich O., Schiffer M. (2023). Partner, Neighbor, Housekeeper and Dimension: 3D versus 2D Glomerular Co-Cultures Reveal Drawbacks of Currently Used Cell Culture Models. Int. J. Mol. Sci..

[166] Sabaté M., Alfonso F., Cequier A., Romaní S., Bordes P., Serra A., Iñiguez A., Salinas P., del Blanco B.G., Goicolea J. (2019). Magnesium-Based Resorbable Scaffold Versus Permanent Metallic Sirolimus-Eluting Stent in Patients With ST-Segment Elevation Myocardial Infarction: The MAGSTEMI Randomized Clinical Trial. Circulation.

[167] Jiang Y., Sun S.-J., Zhen Z., Wei R., Zhang N., Liao S.-Y., Tse H.-F. (2021). Myocardial repair of bioengineered cardiac patches with decellularized placental scaffold and human-induced pluripotent stem cells in a rat model of myocardial infarction. Stem Cell Res. Ther..

[168] Kojima H., Ishii T., Fukumitsu K., Ogiso S., Tomofuji K., Oshima Y., Horie H., Ito T., Wakama S., Makino K. (2023). In Vivo Regeneration of Tubular Small Intestine With Motility: A Novel Approach by Orthotopic Transplantation of Decellularized Scaffold. Transplantation.

[169] Vriend L., Sinkunas V., Camargo C.P., van der Lei B., Harmsen M.C., van Dongen J.A. (2022). Extracellular Matrix-Derived Hydrogels to Augment Dermal Wound Healing: A Systematic Review. Tissue Eng. Part B: Rev..

[170] Marvin J.C., Mochida A., Paredes J., Vaughn B.R., Andarawis-Puri N. (2022). Detergent-Free Decellularization Preserves the Mechanical and Biological Integrity of Murine Tendon. Tissue Eng. Part C Methods.

[171] He P., Yang G., Zhu D., Kong H., Corrales-Ureña Y.R., Ciacchi L.C., Wei G. (2022). Biomolecule-mimetic nanomaterials for photothermal and photodynamic therapy of cancers: Bridging nanobiotechnology and biomedicine. J. Nanobiotechnol..

[172] Devillard C.D., Mandon C.A., Lambert S.A., Blum L.J., Marquette C.A. (2018). Bioinspired Multi-Activities 4D Printing Objects: A New Approach Toward Complex Tissue Engineering. Biotechnol. J..

[173] Sato K., Kawasaki A., Karuo Y., Tarui A., Kawai K., Omote M. (2020). Synthesis of new fluorescent molecules having an aggregation-induced emission property derived from 4-fluoroisoxazoles. Beilstein J. Org. Chem..

[174] Huang H.-K., Hsueh K.-K., Liao Y.-T., Wu S.-H., Chou P.-H., Yeh S.-H., Wang J.-P. (2023). Multilineage differentiation potential in the infant adipose- and umbilical cord-derived mesenchymal stem cells. J. Chin. Med. Assoc..

[175] Marone R., Landmann E., Devaux A., Lepore R., Seyres D., Zuin J., Burgold T., Engdahl C., Capoferri G., Dell’aglio A. (2023). Epitope-engineered human hematopoietic stem cells are shielded from CD123-targeted immunotherapy. J. Exp. Med..

[176] Wang C.-H., Huang Y.-F., Shyu W.-C., Jeng L.-B., Liu S.-P. (2023). Cbx7 promotes the generation of induced pluripotent stem cells. Regen. Ther..

[177] Singh N.K., Han W., Nam S.A., Kim J.W., Kim J.Y., Kim Y.K., Cho D.-W. (2020). Three-dimensional cell-printing of advanced renal tubular tissue analogue. Biomaterials.

[178] Wilems T., Vardhan S., Wu S., Sakiyama-Elbert S. (2019). The influence of microenvironment and extracellular matrix molecules in driving neural stem cell fate within biomaterials. Brain Res. Bull..

[179] Zhang N., Lin J., Lin V.P.H., Milbreta U., Chin J.S., Chew E.G.Y., Lian M.M., Foo J.N., Zhang K., Wu W. (2021). A 3D Fiber-Hydrogel Based Non-Viral Gene Delivery Platform Reveals that microRNAs Promote Axon Regeneration and Enhance Functional Recovery Following Spinal Cord Injury. Adv. Sci..

[180] Vazirzadeh M., Azarpira N., Vosough M., Ghaedi K. (2023). Galactosylation of rat natural scaffold for MSC differentiation into hepatocyte-like cells: A comparative analysis of 2D vs. 3D cell culture techniques. Biochem. Biophys. Rep..

[181] Yadav H.O.S., Kuo A.-T., Urata S., Funahashi K., Imamura Y., Shinoda W. (2022). Adsorption characteristics of peptides on ω-functionalized self-assembled monolayers: A molecular dynamics study. Phys. Chem. Chem. Phys..

[182] Franck C.O., Fanslau L., Popov A.B., Tyagi P., Fruk L. (2021). Biopolymer-based Carriers for DNA Vaccine Design. Angew. Chem. Int. Ed..

[183] Giovanniello F., Asgari M., Breslavsky I.D., Franchini G., Holzapfel G.A., Tabrizian M., Amabili M. (2023). Development and mechanical characterization of decellularized scaffolds for an active aortic graft. Acta Biomater..

[184] Kong D., Wang Q., Huang J., Zhang Z., Wang X., Han Q., Shi Y., Ji R., Li Y. (2023). Design and manufacturing of biomimetic scaffolds for bone repair inspired by bone trabeculae. Comput. Biol. Med..

